# Comparative Study of the Durability Behaviors of Epoxy- and Polyurethane-Based CFRP Plates Subjected to the Combined Effects of Sustained Bending and Water/Seawater Immersion

**DOI:** 10.3390/polym9110603

**Published:** 2017-11-12

**Authors:** Bin Hong, Guijun Xian, Hui Li

**Affiliations:** 1 Key Lab of Structures Dynamic Behavior and Control (Harbin Institute of Technology), Ministry of Education, 73 Huanghe Road, Nangang District, Harbin 150090, China; ahqyhb2008@126.com (B.H.); lihui@hit.edu.cn (H.L.); 2 Key Lab of Smart Prevention and Mitigation of Civil Engineering Disasters of the Ministry of Industry and Information Technology, Harbin Institute of Technology, 73 Huanghe Road, Nangang District, Harbin 150090, China; 3 School of Civil Engineering, Harbin Institute of Technology, 73 Huanghe Road, Nangang District, Harbin 150090, China

**Keywords:** epoxy resin, polyurethane, CFRP plates, water, seawater, bending strain, water uptake, in-plane shear strength, tensile properties

## Abstract

In many applications, carbon fiber reinforced polymer (CFRP) composite materials suffer from the combined effects of sustained bending and immersion. In the present study, pultruded epoxy- and polyurethane (PU)-based CFRP plates were studied for their long-term performances, subjected to the combined effects of water/seawater immersion and sustained bending (0%, 30%, and 58% of the ultimate strain). The water uptake and the evolution of the mechanical properties were investigated. In addition, the service lives of the CFRPs were predicted using the Arrhenius method. Generally, the sustained bending led to a decrease in the water uptake, and reduced the mechanical properties. A diffusion model, dividing the cross-section of CFRPs into the “less resin area” and “rich resin area”, was proposed to elucidate the variation of water uptake and mechanical properties. Compared to epoxy-based CFRPs, although PU-based CFRPs possessed a significantly higher water uptake, they exhibited better long-term performances in terms of mechanical properties.

## 1. Introduction

In recent years, carbon fiber reinforced polymer (CFRP) composites with excellent mechanical properties have been widely used in rehabilitation, repair and reinforcement of various civil structures [1,2,3,4,5,6]. During their service lives, they may suffer from certain harsh environments, such as moisture, water, and seawater even at elevated temperatures [7,8,9,10,11]. In addition, CFRP composites may be also subjected to external loadings at the same time, such as sustained tensile stress in pre-stressed reinforcements [3,12] or bending strain [11]. Although many works [9,10,11,13] have been reported on the durability of CFRP composites, it is still a serious concern, especially under combined environment and stress conditions. In the present article, an epoxy resin, a common used resin matrix for CFRP composites, and a polyurethane (PU) resin system, a new emerging matrix for CFRP composites [14], were used for pultruded CFRP plates (CFRPs). A comparative study on the combined effects of water/seawater immersion and sustained bending on epoxy- and PU-based CFRPs was conducted in terms of water uptake, fiber-resin interfacial bond strength, and tensile properties.

As regards the water uptake of a fiber reinforced polymer (FRP) composite, water diffusion mechanisms have been widely studied. Three kinds of water diffusion mechanisms have been proposed, i.e., by resin matrices [15,16], by fiber-matrix interfaces [17], and/or by the voids [18] formed by insufficient wetting fibers or a not effective fiber sizing [19]. In addition, micro-cracks, formed during curing due to the large difference in thermal expansion coefficients of fiber and matrix, have been reported to increase water uptake [20]. During immersion, the micro-cracks may be converted into macro-cracks, and meanwhile some new micro-cracks may form due to swelling [9], resulting in increased water uptake [9,21]. External stresses, including tensile stress and compressive stress, play an important role on the water uptake of a FRP composite [11,22,23,24]. The tensile stress increases the free volume content (the unoccupied volume by polymer molecules, namely “the cavity”) of the resin matrix, and leads to the formation of micro-cracks and even debonding of the fiber-matrix interface, resulting in an increase in the water uptake [23,24]. On the contrary, the compressive stress reduces the free volume content, reducing the water uptake [11]. Therefore, it is a complicated issue to tell how sustained bending affects the water uptake of an FRP, due to the complex stress status inside. Up to now, only a very limited number of papers exist related to the combined effects of immersion and sustained bending on the durability of FRP composites [11,22,23].

Increasing the bending strain leads to variation of the swelling stress, Poison’s ratio, and the free volume content of the resin matrix, which then decreases the water uptake of the CFRPs [11]. A theoretical equation has been utilized to calculate the maximum water uptake of a CFRP subjected to bending [11,22]

(1) $$M_{\infty \varepsilon }=M_{\infty 0}+\sigma_{f}{\left[\frac{1}{2{\left(m+1\right)}^{2}}\frac{V_{b}}{V_{t}}\right]}^{1/m}\left[\frac{1-2v_{12c}}{E_{1c}}-v_{f}\left(\frac{1-2v_{12f}}{E_{1c}}\right)\right]\frac{\rho_{w}}{\rho_{c}}$$

where *M_∞ε_* is the maximum water uptake of the CFRP subjected to bending deformation, *M_∞_*_0_ is the maximum water uptake of the unstrained CFRP, *σ_f_* is the flexural stress, *m* is the Weibull modulus, *V_b_* is the volume fraction of the CFRP in flexure, *V_f_* is the volume fraction of the CFRP in tension, *v*_12*c*_ is the Poisson’s ratio of the CFRP, *v*_12*f*_ is the Poisson’s ratio of the fiber, *E*_1*c*_ is the longitudinal modulus of the CFRP, *v_f_* is the volume content of the fiber reinforcement, *ρ_w_* is the density of water, *ρ_c_* is the density of the CFRP. Note, an obvious difference between theoretical calculation results and the experimental value was found [11]. This may be because the water absorption in the fiber-matrix interfacial zone was not considered.

As regards the short beam shear (SBS) strength of CFRPs, Kafodya et al. [11] gave a reasonable and accurate prediction using a micromechanical model, proposed by Whitney and Drzal [25]:

(2) $$\tau_{xy}=-4.75\mu A_{1}\varepsilon_{0}\bar{x}\exp\left(-4.75\bar{x}\right)$$

where *τ_xy_* is the interfacial shear stress, *ε*_0_ is the applied far-field strain (strain away from the fiber fragmentation point in finite continuous fiber) varies from 1% to 3%, $\bar{x}$ = *x*/*L_c_*, *x* is the position along the fiber-matrix interface, *L_c_* is the fiber fragment critical length measured from the single fiber fragmentation test or samples subjected to each value of absorbed moisture, *μ* and *A*_1_ are defined as

(3) $$\mu =\sqrt{\frac{G_{m}}{E_{1f}-4v_{12f}G_{m}}}$$

(4) $$A_{1}=E_{1f}\left(1-\frac{{\bar{\varepsilon }}_{1f}}{\varepsilon_{0}}\right)+\frac{4KG_{m}v_{12f}}{K+G_{m}}\left[v_{12f}-v_{m}+\frac{\left(1+v_{m}\right){\bar{\varepsilon }}_{m}-{\bar{\varepsilon }}_{2f}-v_{12f}{\bar{\varepsilon }}_{1f}}{\varepsilon_{0}}\right]$$

(5) $$K=\frac{E_{m}}{2\left(2-\frac{E_{2f}}{2G_{2f}}-\frac{2v_{12f}^{2}E_{2f}}{E_{1f}}\right)}$$

(6) $$G_{m}=\frac{E_{m}}{2\left(1+v_{m}\right)}$$

(7) $$L_{c}=\frac{2.375R}{\mu }$$

where *G_m_* is the shear modulus of resin, *E*_1*f*_ is the longitudinal modulus of fiber, ${\bar{\varepsilon }}_{1f}$ is the longitudinal strain of fiber, *v_m_* is the volume fraction of resin, ${\bar{\varepsilon }}_{m}$ is the strain of resin, ${\bar{\varepsilon }}_{2f}$ is the horizontal strain of fiber, *E_m_* is the modulus of resin, *R* is the radius of fiber.

For the tensile properties of CFRPs subjected to the combined immersion and sustained bending, some degradation mechanisms have been proposed [11]. The degradation rate of the tensile strength was not given.

According to the above-mentioned problems, the present study investigated and compared the evolution laws in water uptake, in-plane shear strength (IPSS), and the tensile properties of epoxy- and PU-based CFRPs, subjected to water/seawater immersion at 20 °C or 60 °C together with sustained bending strain levels (0%, 30%, and 58% of the ultimate extensional strain). Scanning electronic microscopy (SEM) observation was conducted to reveal possible mechanisms of water uptake and the variation of the mechanical properties. In addition, the service lives of the CFRPs in some places with typical climate conditions, i.e., Shanghai (China), Tianjin (China), Vancouver (Canada), and Homer (America), were predicted using the Arrhenius method.

## 2. Materials and Methods

### 2.1. Raw Materials and Sample Preparation

In the present study, two resin systems are used as the matrices for pultruded CFRP plates (CFRPs). The first one is polyurethane (PU) resin, a two-component thermosetting polyurethane (Elastocoat^®^ CC6226/101), purchased from BASF Polyurethane Specialties (Shanghai, China). The PU resin system consists of a polyol component and an isocyanate component with a weight ratio of 1:1.259. The second resin matrix is epoxy which is based on the oligomeric prepolymer diglycidyl ether bisphenol-A (DGEBA) epoxy resin, supplied by Nantong Xingchen Synthetic Material Co., Ltd., Nantong, China. The hardener for the epoxy, accelerator and release agent of the epoxy system are methyl hexahydro-phthalic anhydride (MeHHPA), tris (dimethylaminomethyl) phenol (DMP-30), and INT-1890M (AXEL), purchased from Jiaxing Qingyang Chemical Co., Ltd. (Jiaxing, China), Jinan Yisheng Resin Co., Ltd. (Jinan, China), and Beijing Sino Technology Co., Ltd. (Beijing, China) respectively. The weight ratio of epoxy, hardener, accelerator, and release agent is 100:80:2:1.

The carbon fibers used in the present study are the PAN-based carbon fibers (800 tex, 12 K) with an average diameter of 7 μm purchased from Sinopec Co., Ltd. (Shanghai, China). The tensile strength, modulus and elongation at break of the carbon fiber are 3.75 GPa, 210.2 GPa, and 1.78%, respectively.

PU- and epoxy-based CFRP plates with a cross-section of 25 mm × 1.46 mm were pultruded in the Laboratory for FRP Composites and Structures (LFCS), Harbin Institute of Technology (HIT), China. The fiber volume contents of both CFRPs are approximately 69.9%. The mechanical properties, including in-plane shear strength (IPSS) and tensile properties of both CFRPs are listed in Table 1.

### 2.2. Fixture for the Sustained Bending Strain

**Figure 1:**
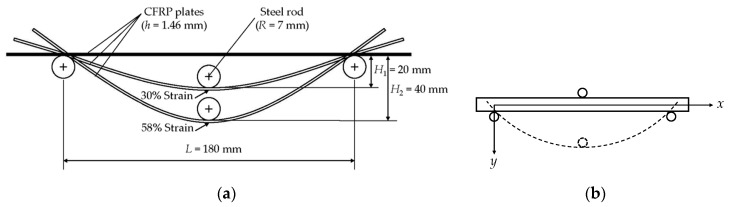
Steel fixture for sustained strain of carbon fiber reinforced polymer (CFRP) plates. (**a**) Schematic sketch, (**b**) the coordination system.

The steel fixture provides two sustained flexural strains for CFRPs as reported previously [11] (Figure 1), which was designed and manufactured in our laboratory. To compare the present results with those from [11], CFRPs in the present study were also assumed to be under pure bending. According to the plane stain theory, the vertical deflection of the specimens under pure bending can be given by [26]

(8) $$v=\frac{M}{2EI}\left(L-x\right)x-\frac{\mu M}{2EI}y^{2}$$

where *v* is the vertical deflection, *M* is the bending moment, *EI* is the material stiffness, *L* is the span, *μ* is the Poisson’s ratio of the specimens, *x* and *y* are established in Figure 1b. The vertical deflection of the neutral axis (*y* = 0) is

(9) $$v=\frac{M}{2EI}\left(L-x\right)x$$

The strain in the *x* direction across the thickness is given by [26]

(10) $$\varepsilon_{xx}=\frac{M}{EI}y$$

where *ε_xx_* is the strain in the *x* direction.

According to Equations (9) and (10), the maximum *x*-direction extensional strain located to the bottom surface at mid span (*y* = *h*/2) is presented as

(11) $$\varepsilon_{xx}=\frac{4vh}{L^{2}}$$

where *h* is the thickness of the CFRP plates.

As shown in Figure 1, two different heights (*H*_1_ and *H*_2_) at the mid span were designed to provide two sustained bending strains. Considering the thickness of the specimens, the two vertical deflections on the neutral axis at the mid span are given by

(12) v=H+h

where *H* is *H*_1_ or *H*_2_ shown in Figure 1a. The ultimate extensional strains of epoxy- and PU-based CFRPs were determined as 1.30% and 1.29%, respectively. According to Equation s(11) and (12), 30% and 58% sustained strain levels are achieved.

According to ASTM D7264 (Standard Test Methods for Flexural Properties of Polymer Matrix Composite Materials), the maximum *x*-direction bending strain located at the bottom surface at the mid-span is calculated by

(13) $$\varepsilon =\frac{6vh}{L^{2}}$$

According to Equations (12) and (13), these two sustained strain levels were determined as 45% and 86%, respectively. Therefore, the 30% and 58% sustained strain levels under pure bending represent the 45% and 86% sustained strain levels under three-point bending, respectively.

### 2.3. Test Methods

#### 2.3.1. Water Uptake

Distilled water and artificial seawater at 20 and 60 °C were used as the immersion media. The artificial seawater was configured without heavy metals according to ASTM D1141 (Standard Practice for the Preparation of Substitute Ocean Water).

**Figure 2:**
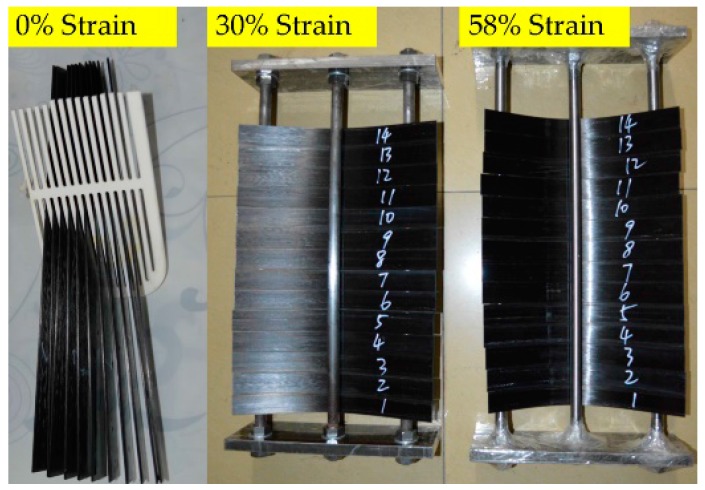
CFRPs under sustained 0%, 30%, and 58% bending strains, respectively. Note, the specimens under sustained 30% and 58% bending strains are placed in the two bending fixtures.

Epoxy- and PU-based CFRP plates were cut into 250 mm longitudinal pieces in the fiber direction. The width and the thickness of these CFRP plates were 25 and 1.46 mm as produced, respectively. All specimens were dried at 60 °C for 2 d in an oven, and then immersed in the designed media and bending deformation (Figure 2).

The immersed specimens were taken out from the water bath and the surface water wiped off using tissue paper, then the masses were measured with an electronic balance with an accuracy of 0.1 mg. The test was performed periodically after immersion, i.e., 2 h, 4 h, 8 h, 16 h, 32 h, 3 d, 5 d, 1 week, 2 weeks, 3 weeks, 1 month, 1.5 months, 2 months, 3 months, 4.5 months and 6 months. A total of 10 samples for each condition were tested and the average values were reported. The mass gain at time *t* (*M_t_*) of a sample is defined as

(14) $$M_{t}=\frac{W_{t}-W_{0}}{W_{0}}\times 100\%$$

where *W_t_* is the mass at time *t*, and *W*_0_ is the initial mass of the sample before immersion.

#### 2.3.2. In-Plane Shear Strength

**Figure 3:**
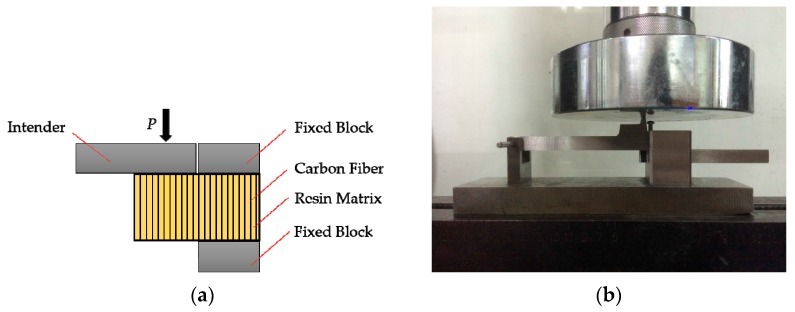
Set-up for in-plane shear strength (IPSS) testing. (**a**) schematic sketch, (**b**) photograph.

**Figure 4:**
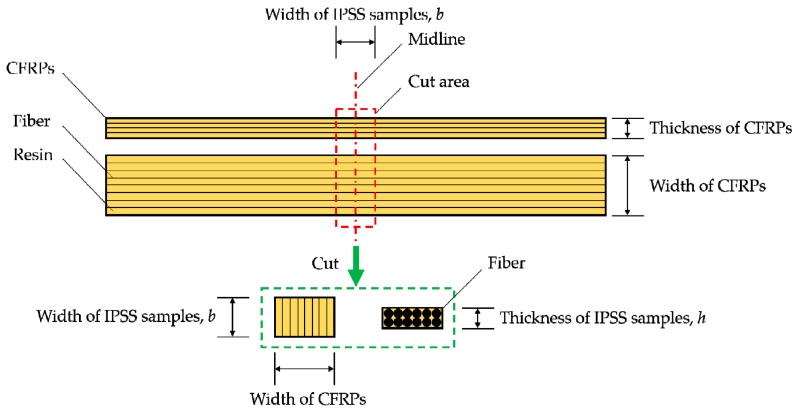
Position of the IPSS testing sample on CFRPs.

The in-plane shear strength (IPSS) [27] test was used to characterize the fiber-resin interfacial bonding strength (Figure 3) of CFRPs. After 3 or 6 months of ageing, IPSSs of CFRPs (25 mm × 10 mm × 1.46 mm) were tested. The testing speed was set as 1 mm/min. For each condition, five samples were repeated, and the average results were reported. Note, the specimens for the IPSS testing were cut from the medium parts of the samples, as shown in Figure 4. The IPSS is calculated by the following equation

(15) $$\mathrm{IPSS}=\frac{P}{hb}$$

where *P* is the failure load, and *h* and b are the thickness and width of a specimen, respectively.

#### 2.3.3. Tensile Properties

After 3 or 6 months of ageing, the tensile properties of the CFRPs (250 mm × 25 mm × 1.46 mm) were tested according to ASTM D 3039 (Standard Test Methods for Tensile Properties of Polymer Matrix Composite Materials). The testing speed was set as 5 mm/min. For each condition, five samples were repeated, and the average results were reported.

#### 2.3.4. SEM Observation

The cross sections and surfaces of the aged CFRP specimens for 6 months were analysed with a scanning electron microscope (SEM) (Quanta 200F, FEI, Hillsboro, OR, USA).

## 3. Results

### 3.1. Water Uptake

**Figure 5:**
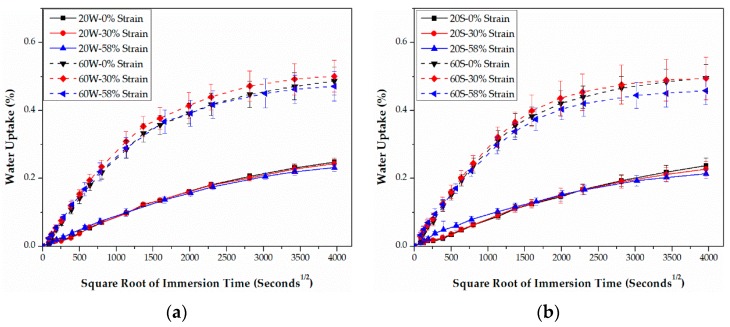
Water uptake curves of epoxy-based CFRPs immersed in distilled water (**a**) and seawater (**b**) for 6 months, respectively.

**Figure 6:**
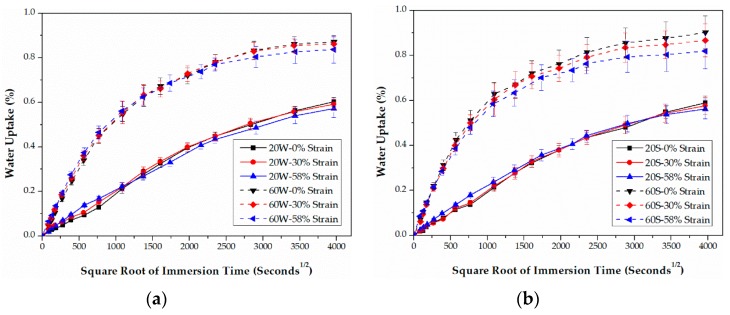
Water uptake curves of PU-based CFRPs immersed in distilled water (**a**) and seawater (**b**) for 6 months, respectively.

Water uptakes of epoxy- and PU-based CFRPs immersed in water/seawater at 20 and 60 °C for 6 months are presented in Figure 5 and Figure 6, respectively. As shown, water uptakes of both CFRPs increase linearly during the initial immersion and then level off. This indicates that these water diffusion behaviors follow the classical Fick’s model [28].

The diffusion coefficients of the samples were calculated by [29]

(16) $$D=\pi {\left(\frac{h}{4M_{m}}\right)}^{2}{\left(\frac{M_{2}-M_{1}}{\sqrt{t_{2}}-\sqrt{t_{1}}}\right)}^{2}$$

where *D* is the diffusion coefficient, *M_m_* is the maximum water uptake (*M*_max_, assumed to be the equilibrium level for the strict Fickian response), *h* is the thickness of CFRPs, *M*_1_ and *M*_2_ is the water uptake at time *t*_1_ and *t*_2_, respectively. The corresponding diffusion parameters of CFRPs are listed in Table 2.

Although the matrices are different, some common rules can be found for both CFRPs. As shown in Figure 5 and Figure 6, increasing the bending strain does not cause noticeable changes in the water uptake in the initial stage of immersion, but leads to significant decreases in the latter stage. This is anomalous for the moderately strained (30% strain level) epoxy-based samples in the high-temperature (60 °C) immersion, which possess almost the highest water uptake during the whole immersion. Compared to the water uptake in water, the water uptake in seawater decreases more distinctly with increasing bending strain during the latter immersion. Nevertheless, as listed in Table 2, the maximum water uptakes (*M_max_*s) in seawater are still very close to that in water. In addition, the diffusion coefficient (*D*) increases with bending strain for both water and seawater immersion. Compared to *D* in water, *D* in seawater is significantly higher. The only exception is for the unstrained epoxy-based samples at 20°C. Therefore, compared to water immersion, both CFRPs absorb more water in seawater.

The above results are different from the ones reported in [11] on an epoxy-based CFRP. As reported in [11], increasing the bending strain decreased the water uptake during the initial immersion. Furthermore, Kafodya et al. [11] indicated that *M*_max_s of the epoxy-based CFRP in seawater were significantly higher than those in water under 0% and 30% of sustained bending strain. The differences became smaller with increasing bending strain. For water immersion, *D* decreased with the bending strain while the opposite result occurred in seawater. Moreover, *D* in seawater was lower than that in water. Compared to the above results [11], the different water diffusion behaviors in the present study may be due to their different degrees of curing. The glass transition temperatures (*T*_g_) of the epoxy-based CFRP used in [11] is 151.3 °C, which was reported by the authors of Ref 11 in another paper [30]. The epoxy-based CFRP in the present study is 169.9 °C. Note, *T*_g_ of these two CFRPs were tested using the same dynamic mechanical analysis (DMA) machine and *T*_g_s were determined by the peak of tan*δ*. This indicates that the degree of curing of the present epoxy matrix is higher than that in [11].

However, considerable differences in water absorption behaviors between epoxy- and PU-based CFRPs were found. As indicated in Figure 5 and Figure 6, increasing the bending strain reduced the water uptakes of PU-based CFRPs more than that of epoxy-based CFRPs during the latter stage of immersion, especially at high temperature (i.e., 60 °C). In addition, in contrast to epoxy-based CFRPs, PU-based CFRPs exhibit dramatically higher water uptakes in both water and seawater immersion. As listed in Table 2, *M_max_*s of unstrained PU-based CFRPs reach 0.60% and 0.59% in water and seawater at 20°C, respectively. However, for unstrained epoxy-based CFRPs, *M_max_*s reach only 0.49% and 0.50% in water and seawater at 60 °C, respectively. Besides, *D* of PU-based CFRPs is only slightly higher than that of epoxy-based CFRPs at 20 °C, whereas the former is remarkably close to two times the latter at 60 °C. These results indicate that PU-based CFRPs absorb more water when immersed in both water and seawater.

### 3.2. IPSS

**Figure 7:**
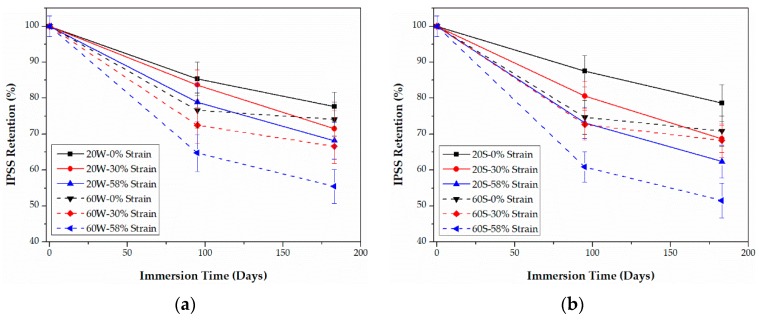
Retention of IPSS of epoxy-based CFRPs immersed in distilled water (**a**) and seawater (**b**) for 6 months, respectively.

**Figure 8:**
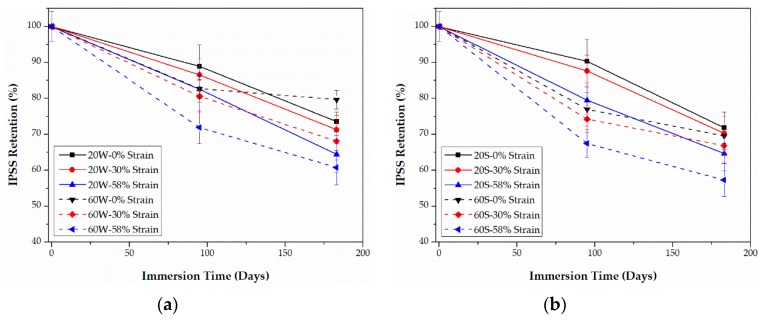
Retention of IPSS of PU-based CFRPs immersed in distilled water (**a**) and seawater (**b**) for 6 months, respectively.

The retention (%) of IPSS values calculated by Equation (17) are plotted in Figure 7 and Figure 8, respectively. As shown, for both epoxy- and PU-based CFRPs, increasing the immersion temperature and bending strain lead undoubtedly to the degradation in IPSS. In addition, IPSS values in seawater are slightly lower than the ones in water in most cases, especially for high temperature of immersions. These results are in line with the trends of the short beam shear (SBS) strength, reported in [11].

(17) $$R_{t}=\frac{P_{t}}{P_{0}}\times 100\%$$

where *R_t_* is the retention of the mechanical properties at immersion time *t*, *P_t_* is the value of the mechanical properties at immersion time *t*, and *P*_0_ is the initial value of the mechanical properties.

As listed in Table 1, the initial IPSS of PU-based CFRPs is obviously higher than that of epoxy-based CFRPs, indicating a durable carbon fiber-PU bonding. However, as shown in Figure 7 and Figure 8, the residual IPSSs of PU-based CFRPs are slightly lower than those of the epoxy-based ones in some cases, e.g., low temperature or none/moderate sustained bending strain. For example, the unstrained epoxy-based CFRPs and PU-based CFRPs immersed in water at 20 °C for 6 months possess 77.7% and 73.6% of the IPSS value, respectively. As the immersion temperature and sustained bending strain increase (more stringent conditions), PU-based CFRPs show better resistance in IPSS. For instance, highly strained (58% strain level) epoxy- and PU-based CFRPs immersed in seawater at 60 °C for 6 months remain at 51.5% and 57.3% of the initial IPSS values, respectively. In view of this, PU-based CFRPs are believed to possess better IPSS in the long run.

### 3.3. Tensile Properties

**Figure 9:**
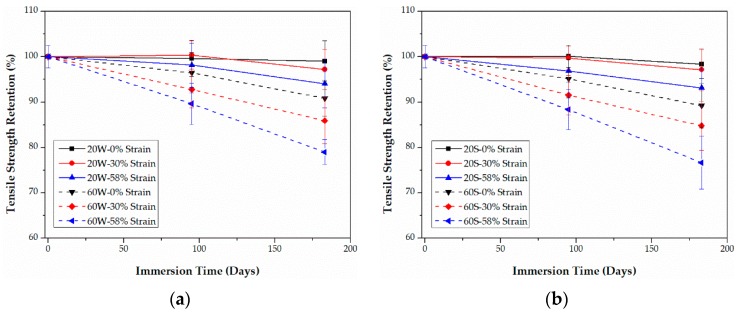
Retention of the tensile strength of epoxy-based CFRPs immersed in distilled water (**a**) and seawater (**b**) for 6 months, respectively.

**Figure 10:**
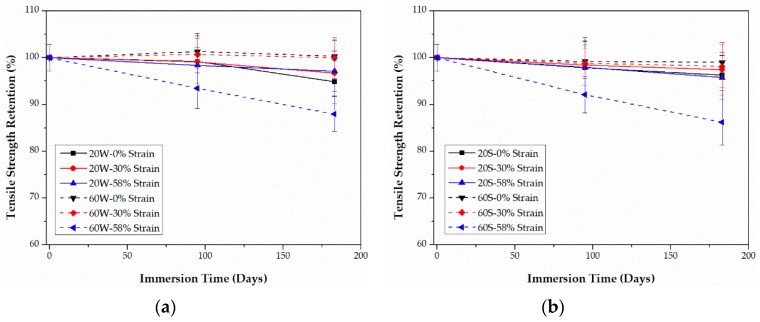
Retention of the tensile strength of PU-based CFRPs immersed in distilled water (**a**) and seawater (**b**) for 6 months, respectively.

**Figure 11:**
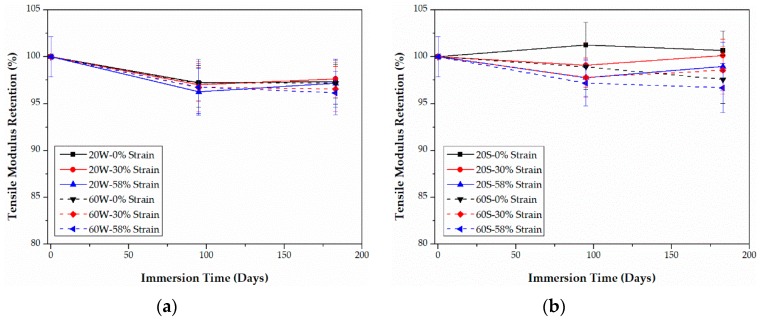
Retention of the tensile modulus of epoxy-based CFRPs immersed in distilled water (**a**) and seawater (**b**) for 6 months, respectively.

**Figure 12:**
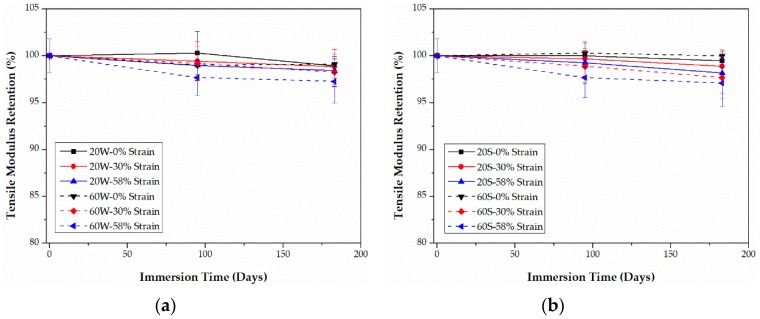
Retention of the tensile modulus of PU-based CFRPs immersed in distilled water (**a**) and seawater (**b**) for 6 months, respectively.

As listed in Table 1, compared with epoxy-based CFRPs, it is noticeable that PU-based CFRPs possess better tensile properties even though their fiber volume contents are the same, which may be ascribed to the better fiber-matrix interfacial bond performance. The retentions (%) of the tensile properties, including strength and modulus, of both CFRPs are plotted as functions of time in Figure 9, Figure 10, Figure 11 and Figure 12, respectively. The tensile elongation at break is not given in the present article since its evolution law is similar to that of the tensile strength [13,31].

As shown in Figure 9 and Figure 10, the tensile strength of both CFRPs in water and seawater decreases with the bending strain, except for PU-based CFRPs at 20 °C. In the latter case, the tensile strength does not show an obvious change with the immersion time (Figure 10). In addition, immersion in seawater leads to more degradation in the tensile strength than that in water in most cases. It is interesting that PU-based CFRPs exhibit a considerable excellent retention in tensile strength (Figure 10). On the one hand, as the immersion temperature increases, the tensile strength of PU-based CFRPs under 0% or 30% of bending strain increases anomalously and even exceeds its initial value slightly, which is contrary to epoxy-based CFRPs. On the other hand, in contrast to PU-based CFRPs, epoxy-based CFRPs immersed in water/seawater at 20 °C show also a slight degradation in tensile strength, which is in line with the results reported in [11]. According to Figure 9, 90.8% and 89.2% of the residual strengths are found for the unstrained epoxy-based CFRP samples immersed in water and seawater at 60 °C for 6 months, respectively, while almost no degradation for the unstrained PU-based CFRP samples. Although serious degradation in the tensile strength occurred for the highly strained (58% of strain level) PU-based CFRPs at 60 °C, only 12.1% and 13.8% of degradation was found in water and seawater, respectively. On the contrary, the highly strained epoxy-based CFRPs degraded by 21.0% and 23.3% of tensile strength in water and seawater at 60 °C, respectively. Therefore, compared to epoxy-based CFRPs, PU-based CFRPs possess a better long-term performance in tensile strength under water/seawater immersion and even sustained bending in the long run.

As shown in Figure 11 and Figure 12, increasing the bending strain causes a decrease in the tensile modulus for both CFRPs in water and seawater. Contrary to the tensile strength, the tensile modulus in seawater is slightly higher than that in water, especially for epoxy-based CFRPs. Nevertheless, the tensile modulus degrades much less than the tensile strength. The highest degradation in the tensile modulus of PU-based CFRPs is approximately 2.9% which is slightly lower than that (equal to 3.9%) for epoxy-based CFRPs. The negligible variation in the tensile modulus of CFRPs is in line with many references [13,22,23].

## 4. Discussion

### 4.1. Mechanism in Water Uptake

**Figure 13:**
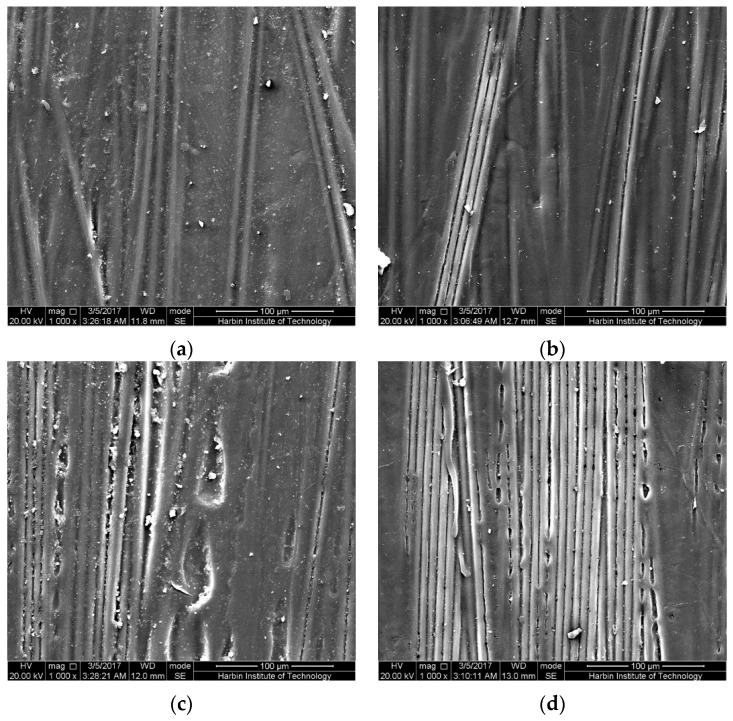
The bottom surfaces in the mid-span region of the epoxy-based CFRP samples after 6-month water immersion at 20 °C: (**a**) controlled samples, (**b**) the unstrained samples, (**c**) samples under 30% strain level, (**d**) samples under 58% strain level.

**Figure 14:**
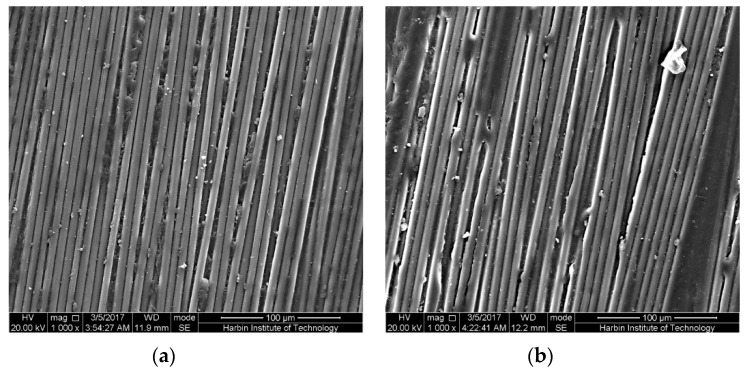
The bottom surfaces in the mid-span region of the epoxy-based CFRP samples after 6-month water immersion at 60 °C: (**a**) the unstrained samples, (**b**) samples under 58% strain level.

**Figure 15:**
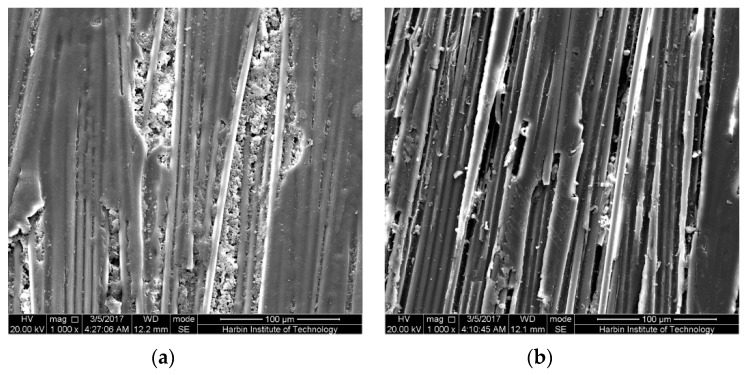
The bottom surfaces in the mid-span region of the epoxy-based CFRP samples subjected to 58% of strain level and seawater immersion for 6 months: (**a**) 20 °C, (**b**) 60 °C.

The bottom surfaces, subjected to tensile stress, of the epoxy-based CFRP samples in the mid-span regions after 6-month water immersion at 20 °C are shown in Figure 13. As shown, as the bending strain increases, more obvious corrosion, namely matrix shedding, occurred. This is responsible for the reduction of the water uptake with the bending strain during the latter immersion. It is worth noting that slight corrosion in the unstrained samples was found, suggesting that the corrosion may be attributed to the hydrolysis of the epoxy matrix. As believed, the increased bending strain leads to more water absorption, and then promotes the hydrolysis of the resin matrix. In addition, the transverse shear stress produced from the sustained bending strain may be also responsible for the increase of the corrosion on the CFRP surface. As the immersion temperature increases, corrosion increases (Figure 14), resulting in a remarkable reduction of the water uptake at 60 °C with high bending strain. This is more obvious for the CFRP samples in corrosive seawater (Figure 15).

**Figure 16:**
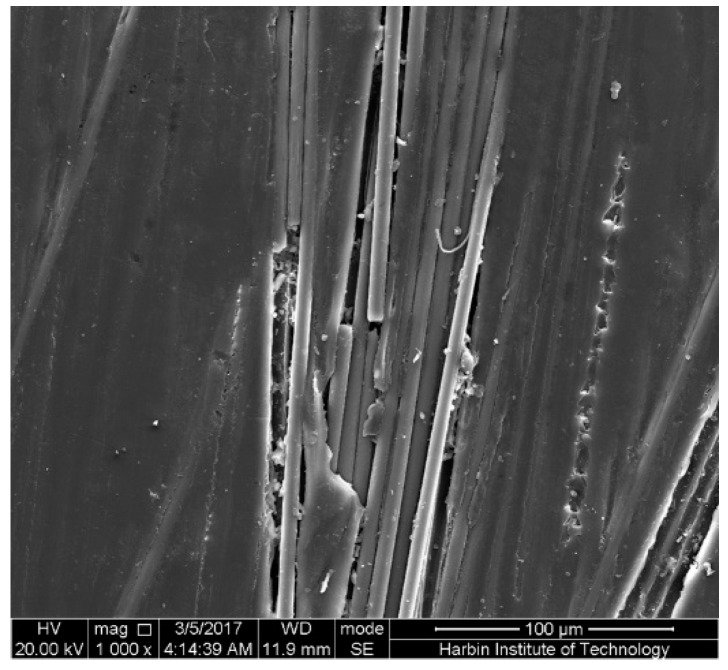
The top surface in the mid-span region of the epoxy-based CFRP samples subjected to 58% strain level and seawater immersion at 60 °C for 6 months.

The top surface, subjected to compressive stress, of the epoxy-based CFRP samples in the mid-span region, subjected to the harshest environment (58% strain and seawater at 60 °C) for 6 months is shown in Figure 16. As found, there is only a slight corrosion on the compressive surface, similar to the unstrained samples. This indicates that the compressive surface of the CFRP samples is almost not affected by the increasing bending strain. The slight corrosion on the compressive surface may be ascribed to the hydrolysis of the epoxy. For PU-based CFRPs, similar SEM pictures were obtained. Accordingly, the tensile stress region of CFRPs plays a crucial role on the water uptake.

**Figure 17:**
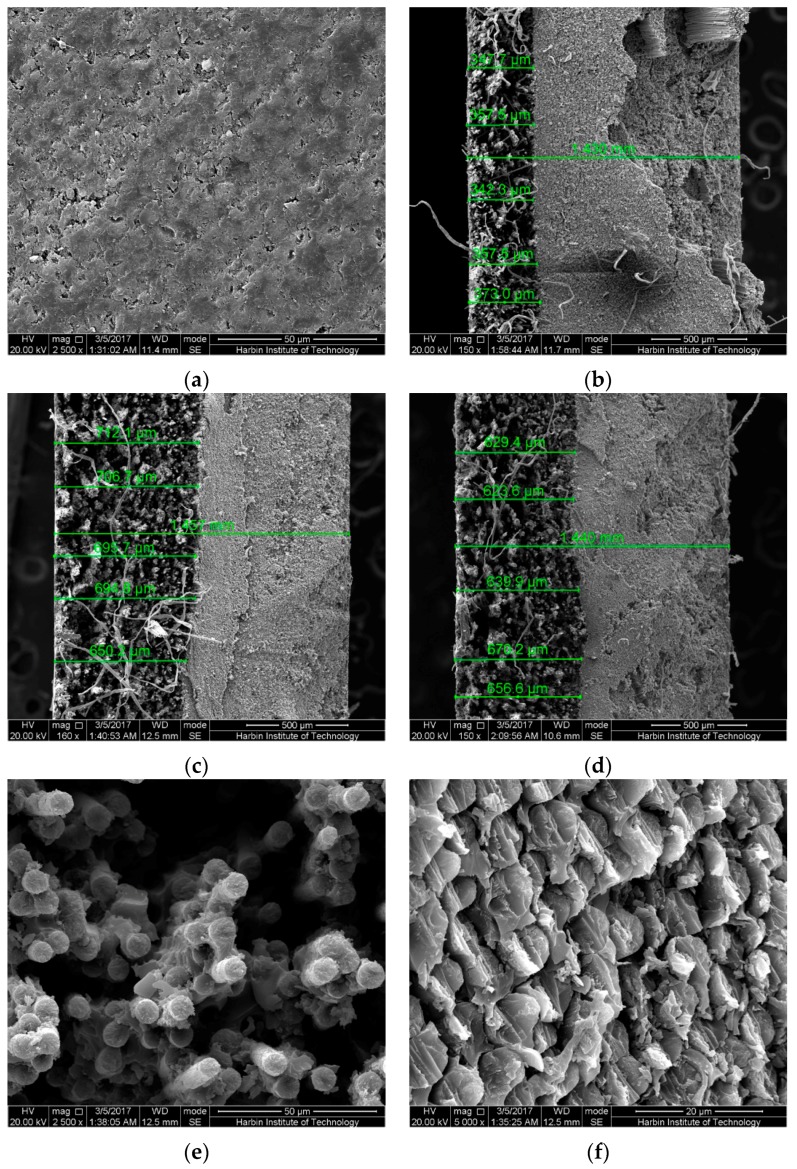
The cross-section in the mid-span region of the broken epoxy-based CFRP samples under 58% of strain level for 6 months: (**a**) controlled samples, (**b**) seawater at 20 °C, (**c**) seawater at 60 °C, (**d**) water at 60 °C, (**e**) tension area in water at 60 °C, (**f**) compression area in water at 60 °C.

**Figure 18:**
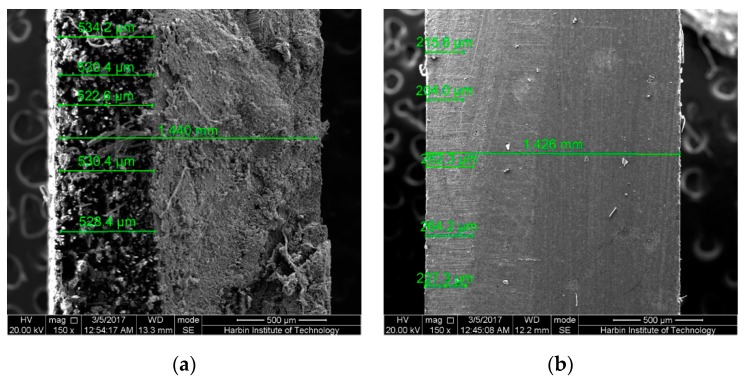
The cross-section in the mid-span region of the PU-based CFRP samples under 58% strain level for 6 months: (**a**) broken samples in seawater at 60 °C, (**b**) unbroken samples in seawater at 60 °C.

**Figure 19:**
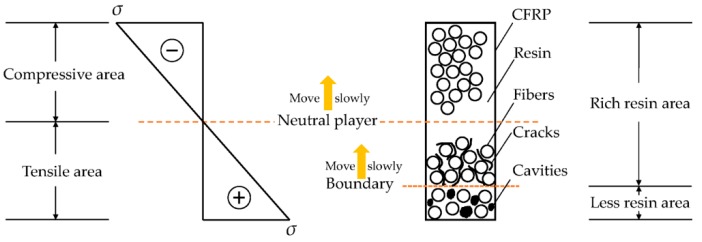
Schematic sketch of the effects of sustained bending on CFRPs immersed in water/seawater in the long run.

It is noteworthy that several highly strained samples after 6-month immersion were broken in the mid-span region due to the uneven force along the width direction while taking the samples out from the bending fixture. The cross-sections in the mid-span regions of these samples were also observed by SEM as shown in Figure 17 and Figure 18. As shown, for both broken epoxy- and PU-based CFRP samples, there is an obvious boundary on the cross-section. The left area (Figure 17e), defined as “less resin area”, shows that almost no resin matrix attaches to the carbon fibers and most of the resin matrix has fallen off, resulting in cavities. This is also responsible for the decrease of the water uptake with increasing bending strain in the latter stage of immersion. The right area (Figure 17f), defined as “rich resin area”, shows that there is still a lot of matrix around the fibers. Nevertheless, a great number of cracks or debonding areas between the fibers and matrix appear in the “rich resin area” near to the boundary, resulting in the increase of water uptake. The schematic sketch of these mechanisms is plotted in Figure 19.

As the immersion temperature increases (Figure 17b,c), the boundary between “less resin area” and “rich resin area” moves to the right, implying that the “less resin area” has expanded with increasing immersion temperature. As investigated, the ratios of the “less resin area” (= the area of “less resin area”/the area of the cross-section) of the broken epoxy-based CFRP samples in seawater at 20 and 60 °C for 6 months are 24.9% and 47.5%, respectively. This is responsible for the slightly larger decrease of the water uptake with increasing bending strain at high temperature of immersion. In contrast to the CFRP samples immersed in seawater, the broken epoxy-based CFRP sample (58% strain in water, 6 months) shows a slightly lower ratio (approximately 44.7%). This verifies again that both epoxy- and PU-based CFRPs are more vulnerable to seawater. For the broken PU-based CFRP sample subjected to seawater immersion at 60 °C for 6 months, the ratio is only 36.8% (Figure 18a), significantly lower than the broken epoxy-based CFRP sample, indicating the excellent carbon fiber-PU interfacial bond. Therefore, compared to PU-based CFRPs, epoxy-based CFRPs are more vulnerable to the combined effects due to serious hydrolysis of the epoxy matrix [32,33] and relatively lower interfacial bond.

Although these broken samples did not break normally, it is believed that all the highly strained samples will break like these broken samples in the long run. As shown in Figure 18b, only a very narrow (16.5%) and weak “less resin area” with almost no resin matrix falling off appears in the highly strained epoxy-based CFRP samples (seawater at 60 °C, 6 months). Therefore, the ratio of the “less resin area” and the degree of shedding increase gradually with increasing immersion time for both epoxy- and PU-based CFRP plates. Furthermore, the tensile stress and compressive stress, including their values and distributions, change slowly with increasing immersion time, exhibiting that the neutral layer (stress is equal to zero) moves to the compressive area (Figure 19). These mechanisms are also illustrated in Figure 19. However, these mechanisms are contrary to the previous assumption that the CFRP specimens are relived of internal tensile and compressive stresses with time after the stress relaxation finished [11].

In summary, during the latter stage of immersion, the decrease of the water uptake of CFRPs with increasing bending strain is ascribed to the shedding of the resin matrix on the surface and in the “less resin area”. The decrease of the water uptake for the seawater immersion is ascribed to more shedding of matrix. The increments of the diffusion coefficients (*D*) with increasing bending strain can be also ascribed to the combined effects, e.g., the increase of the free volume in the matrix, the formation of micro-cracks, and the weak “less resin area”. The PU matrix possesses a unique group, namely –NH–COO– [34], in which the –NH– group can form more hydrogen bonds with water molecules [35], resulting in considerably higher diffusion parameters (water uptake and *D*).

### 4.2. Degradation Mechanism in Mechanical Properties

After 6 months of immersion, the unstrained PU-based CFRP samples were dried in an oven at 60 °C until reaching mass equilibrium. The residual IPSSs of the dried PU-based samples in water at 20 and 60 °C recovered to 92.8% and 100.4% from 73.6% and 79.7%, respectively. For the seawater immersion at 20 and 60 °C, the residual IPSSs of the dried PU-based samples returned only to 88.8% and 95.2% from 71.9% and 69.6%, respectively. On one hand, the reversible degradation in IPSS of both unstrained CFRPs is mainly attributed to the plasticization effect [32]. The plasticization effect is caused by the water-absorbed interrupting some Van der Waals forces and hydrogen bonds, resulting in an increase of molecular mobility and further decrease of IPSS. It is interesting that the CFRP samples subjected to high immersion temperature possess a considerably high recovery rate in IPSS, especially for water immersion, which may be caused by the stronger postcuring effect [11]. On the other hand, the irreversible degradation in IPSS may be due to the formation of micro-cracks caused by swelling. For the strained samples, the formation and strengthening of the shedding of matrix, and the appearance of micro-cracks near to the boundary in the “rich resin area” result in the decrease of IPSS, which is illustrated fully in Figure 19. As mentioned above, in contrast to epoxy-based CFRPs, PU-based CFRPs showed remarkably narrower “less resin area” in seawater under high bending strain level. This is believed to be responsible for the higher residual IPSS for the PU-based CFRP samples immersed at high temperature under high bending strain level.

Generally, the interfacial bond strength represented by IPSS in the present study plays a considerable role in the tensile strength of FRP [36,37]. Therefore, the decrease of IPSS is responsible for the degradation of the tensile strength of these CFRP samples. However, the interfacial bond strength is not only the unique factor affecting the tensile strength of CFRP in the short term. For instance, the unstrained or moderately strained PU-based CFRP samples subjected to immersion at 60 °C showed a significantly higher tensile strength than the ones subjected to immersion at 20 °C. This is believed to be caused by the postcuring effect [11]. Therefore, for a resin matrix cured inadequately, the tensile properties of CFRPs immersed in water/seawater at high temperature even under sustained bending strain may present an abnormal increment due to postcuring. With regard to the slight degradation in tensile modulus, this is because the tensile modulus of the CFRP is determined mainly by the carbon fibers which do not degrade in water/seawater.

### 4.3. Life Prediction of Tensile Strength

In this article, the Arrhenius method [13,38] was utilized to predict the service life of the tensile strength of both epoxy- and PU-based CFRPs in water/seawater under various sustained bending strains. A fundamental assumption was proposed that the single dominant degradation mechanism (debonding at the fiber-matrix interface [39]) does not change regardless of the immersion time and temperature even under various sustained bending strains, but the degradation rate increases with the immersion temperatures. The degradation rate is expressed as follows [40]

(18) $$k=A\exp\left(-E_{a}/RT\right)$$

where *k* is the degradation rate (1/time), *A* is the constant of material in degradation process, *E_a_* is the activation energy (kJ/mol) representing the energy required for degradation, *R* is the universal gas constant (8.314 J·mol^−1^·K^−1^), *T* is temperature in Kelvin. Equation (12) can be transformed as:

(19) $$\frac{1}{k}=\frac{1}{A}\exp\left(E_{a}/RT\right)$$

(20) $$\ln\left(\frac{1}{k}\right)=\frac{E_{a}}{R}\frac{1}{T}-\ln\left(A\right)$$

The inverse of *k* is expressed as the time required for a material property to reach a given value.

Since the fiber-matrix interfacial debonding occurred during the degradation of CFRPs (Figure 17), the exponential degradation model [38] was used, which is defined as

(21) Y=100exp(−t/τ)

where *Y* is the tensile strength retention (%), *t* is the immersion time (Day), and *τ* is a constant.

The values of *τ* and the correlation coefficient (*R*^2^) of both CFRPs are listed in Table 3 by fitting Figure 9 and Figure 10 using Equation (21). It is noteworthy that the fitting parameters of the free/moderately strained PU-based CFRP samples cannot be obtained since the strong postcuring effect results in higher tensile strength at 60 than at 20 °C. Moreover, the small values of *R*^2^ in a few cases are also caused by postcuring. Most of the *R*^2^s are more than 0.97. As shown in Equation (21), the immersion time (*t*) required for the tensile strength retention to reach a given value (*Y_t_*) can be calculated theoretically. The reduction of the tensile strength retention at time *t* is defined as *ΔY_t_* (equal to 100% minus *Y_t_*). The immersion time (*t*) can be also expressed as *ΔY_t_*/*k*. Thus, 1/*k* is equal to *t*/*ΔY_t_*. Therefore, Equation (20) can be transformed as

(22) $$\ln\left(t\right)=\frac{E_{a}}{R}\frac{1}{T}+\ln\left(\Delta Y_{t}\right)-\ln\left(A\right)$$

(23) $$\ln\left(t\right)=\frac{E_{a}}{R}\frac{1}{T}+B$$

where *B* is equal to ln(*ΔY_t_*) minus ln(*A*). As shown in Equation (23), *E_a_* can be obtained by fitting the ln*t* vs. 1/*T* curves. To calculate *E_a_* accurately, the times required for the tensile strength retention to reach 60%, 70%, 80%, and 90% were adopted [38]. The average *E_a_*s are listed in Table 4.

According to Equation (18), the time-shift factor (TSF) for the tensile strength retention to reach the same value *Y* at temperatures *T*_1_ and *T*_0_ can be calculated as

(24) $$\mathrm{TSF}=\frac{t_{0}}{t_{1}}=\frac{\left(1-Y\right)/k_{0}}{\left(1-Y\right)/k_{1}}=\frac{k_{1}}{k_{0}}=\frac{A\exp\left(-E_{a}/RT_{1}\right)}{A\exp\left(-E_{a}/RT_{0}\right)}=\exp\left[\frac{E_{a}}{R}\left(\frac{1}{T_{0}}-\frac{1}{T_{1}}\right)\right]$$

TSF with reference temperatures *T*_0_ equal to 17.0, 13.7, 10.3, and 3.5 °C, representing the annual mean temperatures of Shanghai (China), Tianjin (China), Vancouver (Canada), and Homer (America), respectively, are listed in Table 5 and Table 6. It is worth noting that the effects of freeze-thaw cycle on the tensile strength of CFRPs are not considered in this article. The considered main factor for the selected cities is the annual mean temperature.

**Figure 20:**
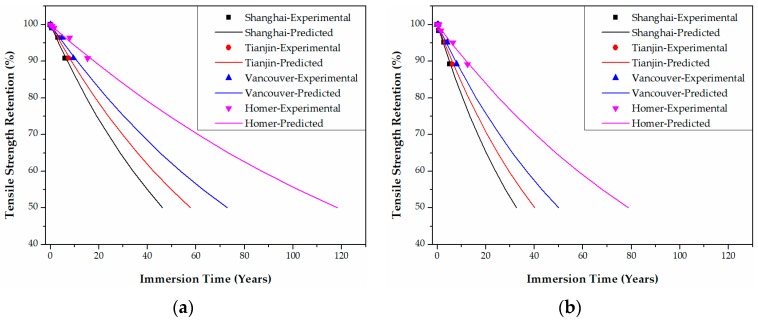
Life prediction of the tensile strength for unstrained epoxy-based CFRPs immersed in distilled water (**a**) and seawater (**b**) in the four cities, respectively.

**Figure 21:**
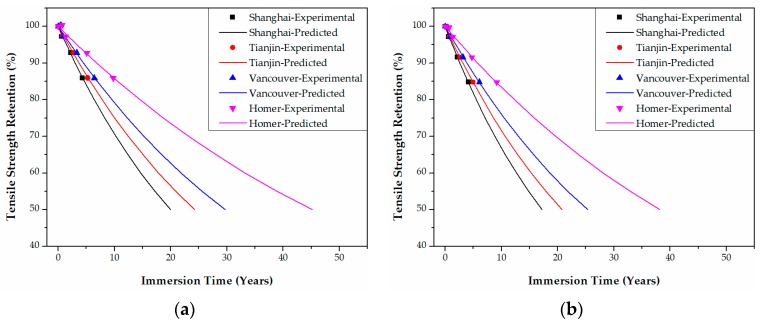
Life prediction of the tensile strength for epoxy-based CFRPs (30% strain) immersed in distilled water (**a**) and seawater (**b**) in the four cities, respectively.

**Figure 22:**
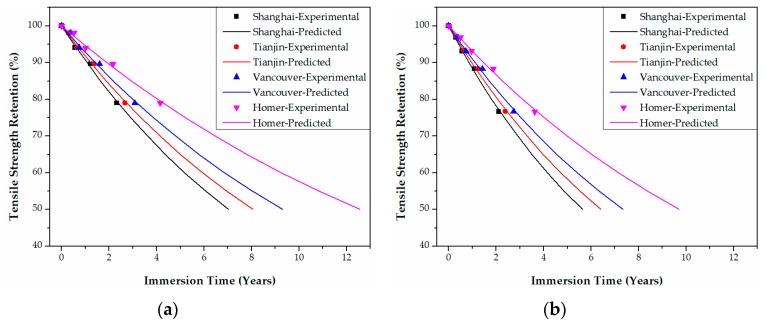
Life prediction of the tensile strength for epoxy-based CFRPs (58% strain) immersed in distilled water (**a**) and seawater (**b**) in the four cities, respectively.

**Figure 23:**
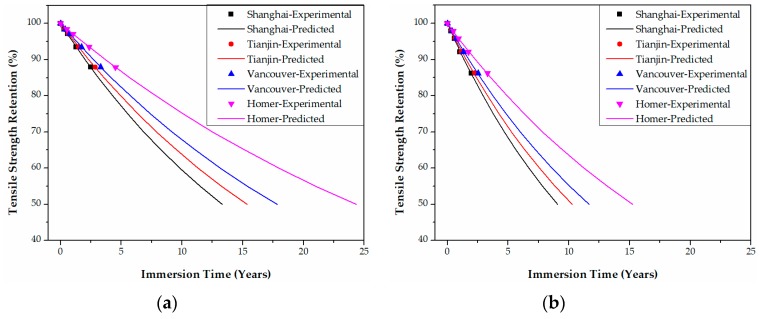
Life prediction of the tensile strength for PU-based CFRPs (58% strain) immersed in distilled water (**a**) and seawater (**b**) in the four cities, respectively.

In Shanghai in China, for example, the tensile strength retention data of epoxy-based CFRPs in Figure 11 are transformed into those of Figure 20 by multiplying the immersion time at 20 °C and 60 °C with the corresponding TSF values. The predicted curve as a function of the immersion time (years) at Shanghai can be obtained by fitting Equation (21) to the data in Figure 20. Following these steps, the predicted curves of both epoxy- and PU-based CFRPs for the four cities were obtained as in Figure 20, Figure 21, Figure 22 and Figure 23, respectively. It is noteworthy that the data in Figure 20, Figure 21, Figure 22 and Figure 23 are obtained from TSF values (Table 5 and Table 6), which are exact experimental data. The fitting parameters *τ* (*R*^2^ ≥ 0.98) of the predicted curves are listed in Table 7. Therefore, the immersion times required for any tensile strength retention can be obtained easily.

According to the predicted curves, the service lives (years) required for the tensile strength retention to reach 50% of both epoxy- and PU-based CFRPs in various conditions in the four cities are listed in Table 8. As shown, for both CFRPs, the service lives decrease dramatically with the increasing immersion temperature and bending strain. Furthermore, the service lives in seawater possess remarkably lower values than the ones in water. In contrast to epoxy-based CFRPs, PU-based CFRPs possess a better long-term performance in tensile strength with approximately twice that of service lives. However, these predicted service lives should be considered conservative since both CFRPs were immersed directly in water/seawater. The corresponding relationships between the degradation in the accelerated environment in laboratory and that in the actual application environment must be established in subsequent research.

## 5. Conclusions

In the present study, the combined effects of water/seawater immersion (20 or 60 °C) and sustained bending (0%, 30%and 58% of its ultimate tensile strain) on the water uptake, IPSS, and tensile properties for both epoxy- and PU-based CFRPs were investigated. The service lives of the CFRPs plates were predicted at Shanghai, Tianjin, Vancouver and Homer (representing different average temperatures) using the Arrhenius method. Based on the above study, the following conclusions were drawn.

(1) A diffusion model was proposed to reveal the water diffusion mechanism. In the model, the cross section of a CFRP plate is divided into “less resin area” and “rich resin area”. As immersion time increases, the neutral layer (stress = 0) and the boundary between the “less resin area” and “rich resin area” moves slowly to the top surface under the combined effects of immersion and bending. At the same time, cracks and fiber-resin debonding tend to occur in the “rich resin area” near to the boundary.

(2) For epoxy- and PU-based CFRPs, the increase of the bending strain reduced the water uptake due to the shedding of the matrix on the surface and in the “less resin area”. The rate of decrease in the water uptake in seawater was higher than that in water during the latter stage of immersion due to the wider “less resin area” in seawater. Due to those combined effects, the diffusion coefficient increased with the bending strain. In contrast to epoxy-based CFRPs, PU-based CFRPs absorbed a higher content of water since the PU matrix possesses a unique group, namely –NH–COO–, in which the –NH– group can form hydrogen bonds with water molecules.

(3) For both epoxy- and PU-based CFRPs, increasing the immersion temperature and bending strain led to reversible and irreversible degradation in IPSS due to plasticization, micro-cracks, and shedding of the matrix in the “less resin area”, respectively. Wider “less resin area” in seawater caused slightly higher degradation in seawater than that in water. In contrast to epoxy-based CFRPs, PU-based CFRPs due to the better interfacial bond were believed to be responsible for the superior IPSS in the long run.

(4) For both epoxy- and PU-based CFRPs, the tensile strength decreased with the bending strain due to the decrease of IPSS. The tensile strength in seawater was slightly lower than that in water in most cases. In contrast to epoxy-based CFRPs, PU-based CFRPs exhibited a better retention of tensile strength, due to better interfacial bonding. In addition, since the tensile modulus of CFRPs is determined mainly by the carbon fibers, degradation in the tensile modulus for both CFRPs was found to be negligible.

(5) The service lives of both epoxy- and PU-based CFRPs subjected to various conditions were predicted, which were conservative. In contrast to epoxy-based CFRPs, PU-based CFRPs possess a much longer service life under the studied service conditions.

## Figures and Tables

**Table 1:** Mechanical properties of epoxy- and polyurethane (PU)-based carbon fiber reinforced polymers (CFRPs).

Mechanical properties	Epoxy-based CFRPs	PU-based CFRPs
In-plane shear strength (IPSS) (MPa)	75.18 ± 2.15	80.41 ± 3.32
Tensile strength (GPa)	2.15 ± 0.05	2.21 ± 0.06
Tensile modulus (GPa)	165.25 ± 3.54	172.26 ± 3.09
Tensile elongation at break (%)	1.30 ± 0.05	1.29 ± 0.08

**Table 2:** Water diffusion parameters of epoxy- and PU-based CFRPs in water or seawater, respectively.

CFRPs	Parameters	Immersion temperature (°C)	20			60		
		Strain level (%)	0	30	58	0	30	58
Epoxy-based CFRPs	*M*_max_ (%)	Water	0.25	0.24	0.23	0.49	0.50	0.47
		Seawater	0.24	0.23	0.21	0.50	0.49	0.46
	*D* (×10^−8^ mm^2^/s)	Water	4.41	4.55	7.53	13.74	15.62	16.81
		Seawater	3.67	4.63	12.32	15.64	17.78	20.39
PU-based CFRPs	*M*_max_ (%)	Water	0.60	0.59	0.57	0.87	0.86	0.84
		Seawater	0.59	0.57	0.56	0.90	0.87	0.82
	*D* (×10^−8^ mm^2^/s)	Water	3.43	5.20	7.72	20.34	24.31	27.92
		Seawater	4.87	5.29	8.25	30.09	30.08	32.82

**Table 3:** Coefficients of regression equation in Equation (21).

CFRP plates	Strain level (%)	Temperature (°C)	Distilled water		Seawater	
			*τ*	*R*^2^	*τ*	*R*^2^
Epoxy-based CFRPs	0	20	20,013.2	0.97	14,276.4	0.64
		60	2032.9	0.56	1665.1	0.77
	30	20	8873.6	0.93	7668.7	0.99
		60	1219.3	0.97	1100.8	0.99
	58	20	3287.1	0.99	2659.0	0.99
		60	800.0	0.99	709.2	0.99
PU-based CFRPs	58	20	6178.3	0.99	4294.1	0.99
		60	1420.2	0.99	1214.8	0.99

**Table 4:** Coefficients of regression equation in Equation (23).

CFRP plates	Strain level (%)	Distilled water		Seawater	
		*E_a_* (kJ/mol)	*R*^2^	*E_a_* (kJ/mol)	*R*^2^
Epoxy-based CFRPs	0	46.4	0.99	43.6	0.99
	30	40.3	0.99	39.4	0.99
	58	28.7	0.99	26.8	0.99
PU-based CFRPs	58	29.9	0.99	25.6	0.99

**Table 5:** Time-shift factors of both epoxy- and PU-based CFRPs immersed in distilled water.

CFRP plates	Strain level (%)	Temperature (°C)	Shanghai 17.0 °C ^a^	Tianjin 13.7 °C ^a^	Vancouver 10.3 °C ^b^	Homer 3.5 °C ^c^
Epoxy-based CFRPs	0	20	1.2	1.5	1.9	3.1
		60	12.0	15.0	18.9	30.7
	30	20	1.2	1.4	1.8	2.7
		60	8.6	10.5	12.8	19.5
	58	20	1.1	1.3	1.5	2.0
		60	4.6	5.3	6.1	8.3
PU-based CFRPs	58	20	1.1	1.3	1.5	2.1
		60	4.9	5.7	6.6	9.0

Note: “^a^”, “^b^” and “^c^” represent the annual mean temperatures of corresponding cities which are derived from the respective authoritative organization as follows: “^a^”: http://www.stats.gov.cn/tjsj/ndsj/2016/indexch.htm (Statistical Data for 2015); “^b^”: http://www.holiday-weather.com/vancouver/averages/ (accessed 31 October 2017); “^c^”: https://www.currentresults.com/Weather/Alaska/average-annual-temperatures.php (accessed 31 October 2017).

**Table 6:** Time-shift factors of both epoxy- and PU-based CFRPs immersed in seawater.

CFRP plates	Strain level (%)	Temperature (°C)	Shanghai 17.0 °C	Tianjin 13.7 °C	Vancouver 10.3 °C	Homer 3.5 °C
Epoxy-based CFRPs	0	20	1.2	1.5	1.8	2.9
		60	10.3	12.7	15.8	24.9
	30	20	1.2	1.4	1.7	2.6
		60	8.2	9.9	12.1	18.3
	58	20	1.1	1.3	1.5	1.9
		60	4.2	4.8	5.5	7.2
PU-based CFRPs	58	20	1.1	1.3	1.4	1.9
		60	3.9	4.5	5.1	6.6

**Table 7:** *τ* (*R*^2^ ≥0.98) of regression equation in Equation (21) for the life prediction curves.

CFRP plates	Media	Strain level (%)	Shanghai 17.0 °C	Tianjin 13.7 °C	Vancouver 10.3 °C	Homer 3.5 °C
Epoxy-based CFRPs	Water	0	24,369.4	30,408.4	38,405.8	62,327.8
		30%	10,527.6	12,757.9	15,623.8	23,784.5
		58%	3704.8	4247.9	4907.2	6618.7
	Seawater	0	17,178.4	21,150.5	26,338.9	41,512.4
		30%	9063.9	10,937.7	13,335.1	20,113.4
		58%	2972.0	3377.7	3865.6	5113.6
PU-based CFRPs	Water	58%	7012.2	8084.8	9394.2	12,824.8
	Seawater	58%	4787.3	5409.8	6154.1	8040.3

**Table 8:** Service lives (years), required for the tensile strength retention to reach 50%, of both epoxy- and PU-based CFRPs in various conditions in the four cities.

CFRP plates	Media	Strain level (%)	Shanghai 17.0 °C	Tianjin 13.7 °C	Vancouver 10.3 °C	Homer 3.5 °C
Epoxy-based CFRPs	Water	0	46.3	57.7	72.9	118.4
		30%	20.0	24.2	29.7	45.2
		58%	7.0	8.1	9.3	12.6
	Seawater	0	32.6	40.2	50.0	78.8
		30%	17.2	20.8	25.3	38.2
		58%	5.6	6.4	7.3	9.7
PU-based CFRPs	Water	58%	13.3	15.4	17.8	24.4
	Seawater	58%	9.1	10.3	11.7	15.3
